# Family planning among undergraduate university students: a CASE study of a public university in Ghana

**DOI:** 10.1186/s12905-019-0708-3

**Published:** 2019-01-17

**Authors:** Fred Yao Gbagbo, Jacqueline Nkrumah

**Affiliations:** Department of Health Administration and Education, University of Education Winneba, Faculty of Science Education, P.O. Box 25, Winneba, Ghana

**Keywords:** Family planning, Undergraduate students, University of Education, Winneba, Ghana

## Abstract

**Background:**

Globally, the rate of unplanned pregnancies among students at institutions of higher education, continue to increase annually despite the universal awareness and availability of contraceptives to the general population. This study examined family planning among undergraduate university students focusing on their knowledge, use and attitudes towards contraception in the University of Education Winneba.

**Methods:**

The study was a descriptive cross-sectional survey using a structured self-administered questionnaire. One hundred undergraduate students from the University of Education Winneba were selected using a multistage simple random sampling technique. A Likert scale was used to assess the attitude of the respondents towards family planning methods.

**Results:**

Findings show that the respondents had a positive attitude towards family planning with an average mean score of about 4.0 using a contraceptive attitude Likert scale. Knowledge of contraception, awareness and benefits however do not commensurate contraceptive use among undergraduate students since availability, accessibility and preference influence usage. Emergency Contraception (Lydia) was reported as easy to get contraceptive, hence the most frequently used contraceptive (31%) among young female students aged 21-24 years who appeared as the most vulnerable in accessing and using contraceptives due to perceived social stigma.

**Conclusion:**

The observation that levels of Family Planning awareness levels do not commensurate knowledge and usage levels calls for more innovative strategies for contraceptive promotion, and Education on the various university campus. The study recommends that public Universities in Ghana should consider a possible curriculum restructuring to incorporate family planning updates. In this regard, a nationwide mixed method study targeting other tertiary institutions including colleges of education in Ghana is required to explore the topic further to inform policy and programme decisions.

**Electronic supplementary material:**

The online version of this article (10.1186/s12905-019-0708-3) contains supplementary material, which is available to authorized users.

## Background

The global incidence of unplanned pregnancies amongst students at higher educational institutions every year continues to increase despite the high awareness and knowledge on regular modern contraceptives and emergency contraceptives among students in higher educational institutions [1, 2]. Despite the immense contraceptive benefits for students in higher educational institutions [3], there is no direct positive correlation between the universal awareness, knowledge and use of contraceptives which challenges global health efforts. The poor utilisation of contraceptives in tertiary institutions is associated with many interrelated factors ranging from personal to institutional setbacks [4]. This eventually contributes to high unplanned pregnancy rates which is estimated to have contributed to about 8 to 30 million annual pregnancies worldwide [5]. Global estimates have also shown that about 210 million pregnancies occur annually across the world. 75 million (or about 36%) of the 210 are unplanned or unwanted pregnancies [6]. Students between 18 and 24 years report the highest rates of unplanned pregnancies in the world’s tertiary institutions [7, 8]. A situation associated with multiple challenges across the world for countries, academic institutions and the individuals involved [9].

Studies in Africa, have generally documented low knowledge and awareness levels of effective contraceptive use amongst higher educational students [10]. Several factors including age, culture, ethnicity, religion, poor access to contraceptive services, peer pressure and lack of partner support were identified as contributing to the non-utilisation of contraceptives in tertiary institutions [11]. In a study amongst 15 to 24 year old South African women, it was estimated that only 52.2% of sexually experienced women are using contraceptives [12]. Because 80% of undergraduate students at higher educational institutions are sexually active, it is important that they have access to safe, accessible and adequate contraceptive services [13].

Although national surveys on family planning [14] have extensively looked at contraceptive uptake in Ghana, little is known about contraceptive up take among students in Ghanaian Universities. This study therefore examines family planning acceptance among students of the University of Education, Winneba in Ghana to compliment national data on family planning.

## Methods

A descriptive cross-sectional study design using a quantitative approach of data collection was adopted. This design was chosen because it fits studies in natural setting, explains phenomena from the view point of persons being studied and produces descriptive data from the respondent own written or spoken words [15].

The study was conducted in the main campus of the University of Education, Winneba. The university was established in 1992 to train middle and top-level manpower for the educational sector of Ghana. It has four main satellite campuses, (Winneba and Ajumako in the Central Region of Ghana, Kumasi, and Mampong campuses in Ashanti Region of Ghana). The Winneba campus has three smaller campuses with five faculties (Faculty of social science education, Faculty of languages, Faculty of science education, Faculty of educational studies and School of creative Arts).

The study population comprised134 ‘non-resident’ undergraduate students of the University of Education Winneba, between ages 17–36 years in 2017 who were registered with an accommodation agent in Winneba that looks for accommodation for students who are unable to obtain university accommodation on campus. This population and age group was selected because anecdotal evidence shows that being a ‘non-resident’ student has the likelihood of making one vulnerable to sexual exploitations whilst seeking accommodation off campus. This age group was considered to be the reproductive age group of the undergraduate students. Because the University only guarantees on campus residential accommodation for only selected first year students, those who do not get the university’s residential accommodation are likely to be victims of sexual exploitations in the Effutu Municipality where the university is situated. This challenge is due to the scarcity of accommodation coupled with the high rent charges for rented accommodation. As per the estimated sample size calculated, a total of one hundred respondents comprising twenty from each of the five faculties were sampled at random to include both male and female students from the various course levels. This was done to ensure a true representation of the student population for the study.

A multistage sampling technique was used to select these respondents for the study. The first stage involved half day orientation of 2 field assistants (male and female) the estimation of the undergraduate students’ population who falls in this category during the period of the study. The second stage involved sample size calculation using an online Raosoft sample size calculator at 95% confidence interval, 5% margin of error and 50% response distribution [16]. In terms of the figures, the sample size *n* and margin of error *E* are given by:

$$ x=Z{\left({}^c/{}_{100}\right)}^2r\left(100-r\right)n={}^{N\ x}/{{{}_{\Big(\left(N-1\right)E}}^2}_{+x\Big)}E=\mathrm{Sqrt}\left[{}^{\left(N-n\right)x}/{}_{n\left(N-1\right)}\right] $$

Where N is the population size (134), R is the fraction of responses that the study is interested in, and Z(c/100) is the critical value for the confidence level c. The estimated number of respondents were then randomly sampled and contacted for participating in the in the third stage of the study. The fourth stage of the study involved distributing the developed questionnaires to consented students.

A Structured Questionnaire (See Additional file 1), designed by the authors was used to solicit responses from respondents. The questionnaire was exploratory in nature with both opened and closed ended questions to help respondents easily share their views. The questionnaire was pre-tested among 20 potential respondents from a different university. The Contraceptive Attitude Likert scales was used to measure attitudes by asking people to respond to series of statements about the topic, in terms of the extent to which they agree or disagree with them. Thus, tapping into the cognitive and affective components of attitudes [17]. The Contraceptive Attitude Scale presented positive and negative statements to elicit for responses that portray participants’ attitudes relating to contraception.

One hundred questionnaires were administered, and all the answers to a particular question were arranged, numbered and responses were coded. The responses were again listed and grouped, putting those with the same code together. Data analysis was done after data had been collected and checked for completeness and accuracy. The Statistical Package for Social Sciences (SPSS) software version 23 was used for data analysis. Frequencies, percentages and bar charts were used to describe the data in multivariable tables.

### Ethics approval and consent to participate

An approval was obtained from the University prior to data collection. Written consent for participation and publication of findings were also obtained from respondents after the purpose, objectives and potential risk and benefits inherent in the study had been explained to them. Prior to the commencement of the study, the research protocol was presented at the bi-weekly academic research seminars of the Faculty of Science Education, University of Education, Winneba. The seminar brought together lectures of the Faculty (equivalent to an ethical review meeting) who critiqued and reviewed the study protocol for ethical suitability and sound methodology. All participants in the study were given the opportunity to ask questions about the study at any stage, and to withdraw from the study at any time. All data collected were kept confidential and data was analysed anonymously to ensure that results were not traceable to individual respondent.

## Results

The overall response rate for the study was 100%. Table 1 presents the background characteristics of respondents. A large number of the respondents were within the age categories of 21 to 24 years and 25 to 28 years. Most of the respondents were single (86.0%) and have no children (86.0%).

**Table 1:** Background characteristics of respondents

Variable	Number (No)	Percentage (%)
Age (in years)		
17–20	9	9.0
21–24	47	47.0
25–28	31	31.0
29–32	4	4.0
33–36	2	2.0
> 36	7	7.0
Marital status		
Married	3	3.0
Cohabitation	5	5.0
Divorced	1	1.0
Single	86	86.0
Wido*w*/widower	1	1.0
Other	4	4.0
Religious affiliation		
Christian	84	84.0
Islamic	12	12.0
Traditional	0	0.0
Other	4	4.0
Gender		
Male	66	66.0
Female	30	30.0
Other	4	4.0
Employment status		
Employed	11	11.0
Unemployed	78	78.0
No response	11	11.0
Number of living children		
0	86	86.0
1	5	5.4
2	1	1.0
> 2	8	8.0
Level in programme		
100	26	26.0
200	44	44.0
300	25	25.0
400	5	5.0
Ethnicity		
Akan	64	64.0
Ewe	6	6.0
Ga-Adangbe	6	6.0
Mole-Dagbani	11	11.0
Guan	3	3.0
Grusi	1	1.0
Others	9	9.0
History of pregnancy		
Ever pregnant	13	13.0
Never pregnant	77	77.0
No response	10	10.0

*Source: Field data 2017*

Table 2 present results of students’ knowledge, information sources and reasons for accepting or not accepting family planning. Family planning awareness and knowledge among students was a key consideration in the study.

**Table 2:** Family Planning Knowledge, Information Sources and Reasons for Family Planning Acceptance among Student

Details of issues investigated	Responses					
	Yes	%	No	%	NR	%
Ever heard of family planning	94	94.0	2	2.0	4	4.0
Can you get pregnant when using withdrawal method?	67	67.0	25	25.0	8	8.0
Is family planning helpful?	61	61.0	25	25.0	14	14.0
Will you recommend FP to a friend or relative?	69	69.0	20	20.0	10	10.0
*Meaning of* family planning						
Don’t know	9	9.0				
Measures to reduce child birth	21	21.0				
Measures to prevent unwanted pregnancy	14	14.0				
Measures to space child birth	17	17.0				
Measures to reduce number of children and unwanted pregnancy	2	2.0				
Measures to reduce unwanted pregnancy, space and reduce childbirth	33	33.0				
Others	4	4.0				
*Total*	*100*	*100*				
Sources of family planning information						
Don’t know	6	6.0				
Television	31	31.0				
Books	14	14.0				
Radio	9	9.0				
Health worker	11	11.0				
Friend	2	2.0				
Social media	27	27.0				
*Total*	*100*	*100*				
Reasons for family planning						
Don’t know	1	1.6				
Help prevent unwanted pregnancies	20	32.8				
FP promote small family size	16	26.2				
FP helps in planning and catering for children	14	23.0				
Prevent STIs	1	1.6				
FP reduce family expenses	5	8.3				
FP reduce over population	2	3.3				
FP reduces school drop outs	1	1.6				
Others	1	1.6				
*Total*	*100*	*100*				
Reasons against family planning						
Don’t know	4	8.0				
Have not used some before	7	28.0				
Negative side effect	5	20.0				
FP is for only married people	1	4.0				
FP can cause conditions such as fibroid	2	8.0				
FP causes premature menopause	1	4.0				
The bible is against FP	2	8.0				
FP causes barrenness	1	4.0				
FP is not reliable, it can fail	2	8.0				
*Total*	*100*	*100*				

*Source: Field data 2017*

About 94% of respondents answered yes to whether they have ever heard about family planning. Although majority (61%) of the respondents believed FP is helpful, about (67.0%) knew that one could get pregnant by relying on the withdrawal method. It appears most students would be committed to family planning uptake if services are made available. This is evident by 69% of them responding in the affirmative when asked whether they will encourage their family or friends to use family planning services in the University.

Having knowledge of family planning does not necessarily translate into utilization since the respondents had varied reasons for and against using family planning. Respondents who were of the view that FP was not helpful (25.0%) had either not used any family planning method before (28.0%) or had ever suffered unpleasant negative side effects (20.0%) following family planning usage or believed the bible is against family planning (2.0%).

**Fig. 1:**
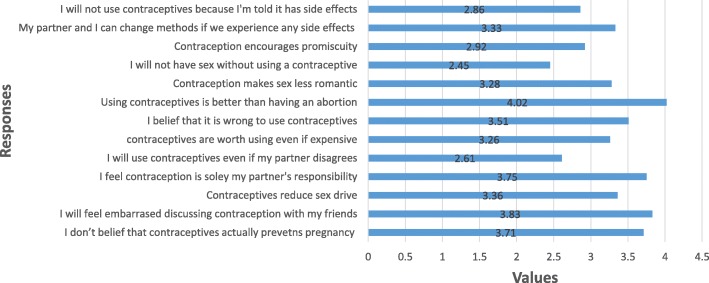
Attitude towards Family Planning

Figure 1 presents respondents’ attitudes towards family planning as estimated using the Contraceptive Attitude Scale. The overall population surveyed had a positive attitude towards family planning (average mean attitude score was about 4.0 out of 5.0).

There were however some divergent responses to the questions relating to contraceptive use. Some of these include:

- ‘I will not have sexual intercourse if no contraceptive method was available’
- ‘I will use contraceptives even if my partner does not want me to use it’
- ‘I will not use contraceptives because they encourage promiscuity’

When the respondents were asked if they have ever used any FP method before, the majority of the respondents (67.0%) mentioned that they had never used any FP method. Regarding availability of family planning service when needed, about 64.0% of the respondents indicated that family planning services are always available in chemical shops and from colleges on campus when needed. About 58% will use FP methods in the future. Regarding information on source of family planning services if required, most of the respondents (85%) knew where to get family planning services in their communities (Table 3). Young Female students aged 21-24 years were the most vulnerable in accessing and using contraceptives due to perceived social stigma relating to a female student buying a contraceptive.

**Table 3:** Source of Family planning services, Utilization and effects of FP methods

Variable	Number			Percentage (%)		
	Yes	No	No response	Yes	No	No response
Have you used any FP method before	29	67	4	29.0	67.0	4.0
Do you always have access to FP in your area?	66	27	7	66.0	27.0	7.0
Do you know where to get FP services by yourself when required?	85	12	3	85	12	3
Have you ever experienced any side effect(s) from any of the FP methods used?	10	69	21	10.0	69.0	21.0
Do you still use FP services?	29	50	21	29.0	50.0	21.0
Do you intend using FP methods in the future	58	31	11	58.0	31.0	11.0

Table 4 documents the various family planning choices and reasons for the choices. About 65.0% of respondents reported that they primarily use contraceptives to prevent pregnancy and usually use a contraceptive before sexual intercourse (34.0%). When asked to select the primary methods of contraception frequently used, Emergency Contraception was the most reported frequently used (51%) contraceptive followed by male condoms (34.0%). Various side effects associated with some FP methods were also reported. Some respondents were of the view that there should be education for students on the risk and benefits of FP methods for effective use. Others believed FP should not be tolerated among students because it can be abused leading to major health problems that could affect studies. Knowledge of contraception, awareness and benefits however do not commensurate contraceptive use among undergraduate students since availability, accessibility, preference and cost of contraceptives hinders use.

**Table 4:** Family planning choices and reasons for the choices

Primary reason for choosing a FP method	Family Planning Choices															
	Condoms		Abstinence		Injectable		Oral contraceptives		implant		Withdrawal	calendar			Emergency contraception	
	No	%	No	%	No	%	No	%	No	%	No	%	No	%	No	%
Number of responses	23	34.0	3	7.0	0	0.0	1	2.6	0	0.0	0	0.0	0	0.0	65	51.0
Easy to get	22	51.2	0	0.0	1	20.0	5	27.8	0	0.0	0	0.0	0	0.0	43	31.0
Easy to use	4	9.3	1	16.7	2	40.0	5	27.8	1	10.0	0	0.0	2	66.7	0	0.0
Effective to use	3	7.0	1	16.6	2	40.0	6	33.3	0	0.0	0	0.0	0	0.0	1	10.0
No side effect	11	25.6	4	66.7	0	0.0	1	5.6	0	0.0	1	10.0	1	33.3	0	0.0

## Discussion

This study examined family planning among undergraduate university students focusing on their knowledge, use and attitudes towards family planning in the University of Education Winneba. The study was a descriptive cross-sectional survey using a structured self-administered questionnaire for data collection. Various findings obtained from the study had reproductive health programme and policy implications. Informal sources of family planning information such as friends, peers and relatives were common information sources for young people [18] but yet prone to misconceptions, distortions and half-truths. Other studies ranked the family (parents, brothers and sisters) as the lowest source of information on sexuality [19–21]. These findings are similar to those reported in the current study that high level of awareness (94.0%) of contraceptives is noted among university students.

An observation that a large number of the respondents were within the age categories of 21 to 24 years and 25 to 28 years of which most (86.0%) were single and have no children (86.0%), is an indication that current university students are relatively young and unmarried. A situation that predisposes them to sexual exploitations and requires knowledge on family planning methods to enable them make informed decision and choices regarding their reproductive intentions. Family planning awareness and knowledge among students was a key consideration in the study. The majority (94%) of respondents indicating that they have ever heard about family planning shows a near universal awareness of family planning methods. This is in line with national reports on family planning awareness in Ghana and a significant departure from many other studies which tended to focus on awareness alone or translate awareness to knowledge [22, 23]. Understanding the methods and benefits of contraception are critical to having motivated users. It has also been noted that motivation is one of the important factors in minimizing failure rates in the utilization of contraception [24]. From previous research findings [25–27] it was established that the most commonly used Family Planning methods among students were short term methods predominantly, condoms, oral contraceptives and withdrawal methods. This confirms finding of other studies that students had little knowledge about effective contraceptive methods [28]. In the current study, a remarkable percentage (25%) did not know that pregnancy could occur when one relying solely on withdrawal method. Also about 21.0% of respondents did not know what oral contraceptive pills do, and some 3% also said oral contraceptive pill prevents Sexually Transmitted Infections (STIs). It was surprising to note in this era of increasing STIs that about 2% of respondents’ from a tertiary institution belief a single condom can be reused many times if washed and dried.

At the tertiary level, one would have expected that all respondents would have known the implications of unprotected sexual intercourse. However the study finding that about (61%) of the respondents believed family planning is helpful implies that there are some other students who don’t belief in family planning hence having unprotected sexual intercourse. Although accessibility to family planning methods on campus in this study was very high (66.0%), results from other similar studies were to the contrary [29, 30]. This therefore suggests that if students know the benefits and how to use contraceptives, they will not experience unwanted pregnancies and its associated consequences of unsafe abortion complications, disruption in academic work and possible death. Contraceptive education is a component of sex education and is one of the proven approaches to prevent risky sexual behaviour and must be introduced on university campuses to guide students’ family planning choices.

Additionally, findings also shows that there are some students about (67.0%) at the university who knew that one could get pregnant by relying on the withdrawal method yet that is their preferred family planning methods. Various studies [31, 32] have explained this observation further by indicating that some adolescents girls feel that a partner’s use of condom suggest that they (the girls) might be classified as unclean, likened to commercial sex workers or seen as engaging in extra-relationship sexual activities if they negotiate for condom use during sexual intercourse. The perception of ‘*I trust my partner so no need for condom use’* further explains the frequency of withdrawal methods being a regular family planning method on campus.

Generally, it appears most students were committed to family planning uptake if services are made available as evident by about 69% of them responding in affirmative when asked whether they will encourage their family or friends to use family planning services in the University. This observation is positive for enhanced family planning service delivery on university campuses to meet the needs of students. Contrary to this observation are those of similar studies which reported that Student frown on invasive family planning methods [33, 34]. The distinction between invasive and non-invasive methods bothers on factors such as availability of method, ease of use and adherence to instructions of a health professional to use the method.

Respondents outlined various sources of family planning information of which television adverts constituted the most reported (31%) source of information. This observation is quite worrying since anecdotal evidence from university campuses shows that majority of student rarely have and watch televisions whist on the various campuses. It will therefore be very important and useful to devise innovative ways of educating students on family planning methods whilst on campus.

A finding that having knowledge of family planning does not necessarily translate into usage is very revealing and of public health importance. As it would have been expected, using a method is the surest way of explaining its relevance. However in this study, respondents who were of the view that family planning was not helpful had never used any family planning method before (28.0%). It is there important to use of family planning satisfied client for contraceptive education and promotion on University campuses to ensure the desired positive results. These are students who are likely to positively influence their sexually active peers on contraceptive use since they are likely to say: ‘*I will not have sexual intercourse if no contraceptive method was available’ or ‘I will use contraceptives even if my partner does not want me to use it’* as reported in the study.

Regarding information on source of family planning services if required, most of the respondents (85%) knew where to get family planning services in their communities. For availability of family planning services when needed, about 64.0% of the respondents indicated that family planning services are always available in chemical shops and from colleges on campus when needed. The obvious indicated sources of contraceptives on campus (i.e., chemical shops and peers) do not provide varying choice of services there by limiting students to short term and less effective family planning methods. It is encouraging noting that about 58% of respondents will use FP methods in future. This is an indication of them understanding the importance of family planning to studies as about 65.0% of respondents reported primarily using contraceptives to prevent pregnancy and usually use a method before sexual intercourse (34.0%) despite the various side effects associated with some FP methods reported.

Knowledge of contraception, awareness and benefits however do not commensurate contraceptive use among undergraduate students since availability, accessibility and preference influence usage. Emergency Contraception (Lydia) was reported as easy to get contraceptive, hence the most frequently used contraceptive (31%) among young female students aged 21-24 years who appeared as the most vulnerable in accessing and using contraceptives due to perceived social stigma. This observation shows that Students always have a unique view on issues especially those in youthful ages. It is therefore important to incorporate their views in family planning programming. The observation that some respondents were of the view that there should be education for students on the risk and benefits of family planning methods for effective use is in the right direction and worth exploring. There are also concerns of values clarification as observed by the findings that some respondents believed family planning should not be tolerated among students because it can be abused leading students to becoming promiscuous or suffering major health problems that will affect their studies.

The following recommendations are therefore being suggested to chart a way forward:

- Public Universities in Ghana should consider a possible curriculum restructuring to incorporate family planning lessons in the academic programme for students to acquire current knowledge in this area. The reproductive health education programs should include the importance of using dual contraceptive methods as a means to prevent HIV transmission and pregnancy, as well as information on how to make an informed decision relating to contraceptive choices.

- The Winneba Municipal Health Directorate should incorporate family planning education on campuses into their public health programs.

- The university health service should also create friendly environment for student to access family planning services and also collaborate with the student body to organise programmes to educate the students on family planning methods.

- The student representative council (SRC) should also make family planning education a part of their programs and in collaboration with the university health services organise free STI testing and family planning counselling at least once yearly.

- A nationwide mixed method study targeting other tertiary institutions particularly colleges of education in Ghana is required to explore the topic further for a national decision on contraceptive security in tertiary institutions in Ghana.

## Conclusions

Findings of this study showed that the awareness of family planning among the students was high. However, levels of contraceptive usage were low and restricted to the short term, Emergency Contraceptives and redrawal methods. The perception by a cross-section of respondents (although by a small group) that condoms can be reused more than once confirms the gross ignorance of contraception practices and the potential risk to STIs and Pregnancy. Additionally, Emergency Contraception (Lydia) being reported as easy to get contraceptive, hence the most frequently used contraceptive (31%) among young female students aged 21-24 years, is an indication that this student population appeared as the most vulnerable in accessing and using contraceptives due to perceived social stigma and must therefore be the prime focus of contraception education and services on the University. The University of Education being a tertiary institution mandated to train teachers, is expected to ensure that its students have accurate and current information on family planning methods relevant to educate others. This is an obvious gap that requires policy decisions at all levels and FP education interventions at the tertiary level of education in Ghana.

## Additional file

Additional file 1: Appendix I-Questionaire. The appendix I contains the structured question developed by the authors and used for data collection in the study. (DOCX 23 kb)

## Abbreviations

FP: Family planning
SPSS: Statistical Package for Social Sciences
STI: Sexually Transmitted Infections
UEW: University of Education Winneba
