# Supplementary material for: Family planning among undergraduate university students: a CASE study of a public university in Ghana
Source: BMC Womens Health. 2019 Jan 17;19:12. doi: 10.1186/s12905-019-0708-3 (PMC6337791; doi:10.1186/s12905-019-0708-3)
Supplement: Supplementary file 1 — Appendix I-Questionaire. The appendix I contains the structured question developed by the authors and used for data collection in the study. (DOCX 23 kb) [file 12905_2019_708_MOESM1_ESM.docx]

# APPENDIX I: QUESTIONNAIRE

This questionnaire is to collect data on (family planning among undergraduate students of the University of Education, Winneba). The outcome of this study will be used to inform policy and programme decisions in the university and also add to the existing stock of knowledge in this area of study. I would be grateful if you could provide me with accurate and detailed information on this survey. The confidentiality of your information is much assured.

**SECTION A: BACKGROUND CHARACTERISTICS OF RESPONDENTS**

1. Sex
2. Male [ ] b. Female [ ]
3. Age in years…………………….
4. Ethnic group ………………….
5. Level in programme

a.100 [ ] b. 200 [ ]

c. 300 [ ] d. 400 [ ]

1. Religion
2. Christian [ ] b. Muslim [ ]

c.Traditionalist[] d. others………......................

1. Are you employed?
2. Yes [ ] b. No [ ]

**[ if No skip to Ques 7]**

1. If yes specify Occupation

…………………………

1. Marital status
2. Married [ ] b. Cohabitation

c. Divorced [ ] d. single [ ]

e. widow/widower [ ]

9. How many children do you have, if any?

a. None [ ] b. 1 [ ] c. 2 [ ]

d. 3 or more [ ]

10. Have you or your partner ever been pregnant before?

a. Yes [ ] b. No [ ]

**SECTION B: KNOWLEDGE ABOUT FAMILY PLANNING METHODS**

1. Have you ever heard about family planning methods/contrceptives?
2. Yes [ ] b. No [ ]
3. If yes from where?
4. Television [ ] b. Books [ ]

c. radio [ ] d. health worker [ ]

e. relative [ ] f. friends [ ]

g. others………………………

1. In your own view what is family planning…………………………………………………………………………………………………………………………………………
2. What does a contraceptive generally do?

a. It prevents ovulation [ ]

1. It prevents implantation of the fertilised ovum [ ]
2. It prevents sexually transmitted diseases [ ]
3. All of the above [ ]
4. I don’t know [ ]
5. How many times can you use a single condom? a. Only once [ ]

b. Twice, if you wash it properly after use [ ] c. Multiple use [ ]

16. Can you get pregnant if you/your partner use the withdrawal method?

a. Yes [ ] b. No [ ]

17. Have you ever used a family planning method?

a. Yes [ ] b. No [ ]

18. If Yes why?

………………………………………………………………………………………………………………………………………

19. If no why not?

………………………………………………………………………………………………………………………………………

20. Will you recommend family planning to a friend or relative?

a. Yes [ ] b. No [ ]

If Yes why?

…………………………………………………………………………………………

If no why not?

…………………………………………………………………………………………

**SECTION C: ATTITUDE TOWARDS CONTRACEPTIVE**

Below are several statements about the use of contraceptives (birth control). We are interested in knowing your opinion about each statement. Using the scale below, please indicate your level of agreement or disagreement with each statement. Keep in mind that there are no right or wrong answers. Also remember that we are interested in your personal opinion. Therefore, we want to know how you feel about these statements and not how you think your family or friends might feel about these statements.

**1 = Strongly agree; 2 = Agree; 3 = Undecided; 4 = Disagree; 5 = Strongly disagree**

21. I believe that it is wrong to use contraceptives. 1……2…….3…….4…….5…....

22. Using contraceptives is much more desirable than having an abortion 1……2…….3…….4…….5……..

23. Contraceptives make sex seem less romantic.

1……2……..3…….4…….5………

24. I would not have intercourse if no contraceptive method was available 1…….2…….3…….4……...5……….

25. I do not believe that contraceptives actually prevent pregnancy

1……2……..3…….4……..5………..

26. I would feel embarrassed buying or discussing contraception with my friends

1…….2……..3…….4……..5……….

27. Contraceptives reduce sex drive. 1……2………3….…4………5….…..

28. I feel that contraception is solely my partner's responsibility so he must buy it.

1……..2……..3………4…….5…………

29. I would use contraceptives even if my partner does not want me to

1…….2……….3………4……..5……….

30. Contraceptives are worth using, even if the monetary cost is high

1……..2………3………4………5………

31. Contraceptives encourage promiscuity.

1…….2……….3……….4………5………

32. If I or my partner experienced negative side effects from a contraceptive method we would use a different method 1……2………3………4……..5…………

33. I will not use contraceptive because I have heard that it has many side effects.

1……2………3………4……..5…………

**SECTION D:-UTILIZATION**

34. Have you ever used any family planning method before?

A. Yes [ ] b. No [ ]

35. Do you have access to family planning methods in your area?

a. Yes [ ] b. No [ ]

36. Where do you access family planning services?

a. Health facilities [ ]

b. Pharmarcy/Chemist shop [ ]

e. others…………………………………

37. What is your primary reason for using contraceptive?

a. pregnancy prevention [ ] b. STI prevention [ ]

c. Others…………………………………

38. If yes, what is your primary method of contraception?

a.Condoms [ ] b. Injectables

c. Oral contraceptives [ ] d. Implant [ ]

e. Intra uterine device [ ] f. Vaginal contraceptives [ ] g. Withdrawl [ ]

h. Calender method [ ] i. Tubal sterilisation [ ] j. Abstinence [ ] k. Vasectomy [ ]

l. Breastfeeding [ ] m. Diaphram [ ] n. emergency contraceptive [ ]

39. What is your primary reason for the above choice?

a. easy to get [ ] b. easy to use [ ]

c. effective to use [ ] d. No side effect [ ]

e.Others………………………………………………………………………………

40. Did you experience any side effect(s) of any of the family planning methods you ever used? a. Yes [ ] b. No [ ]

41. If Yes what is it or are they?

…………………………………………………………………………………………

42. Do you still use family planning service? a. Yes [ ] b. No [ ]

43. When do you use contraceptives?

a. After child birth [ ] b. Before sex [ ] c. after sex [ ] d. Always [ ] e.Have not use one [ ]

44. How often do you have sex?

a. Not at all [ ] b. Yearly [ ]

c. Monthly [ ] d. Fortnightly [ ]

e. Weekly [ ] f. Everyday [ ]

45. How often do you use family planning method?

a. Not at all [ ] b. Yearly [ ]

c. Monthly [ ] d. Fortnightly [ ]

e. Weekly [ ] f. Everyday [ ]

46. Which contraceptive method do you use most frequently?-------------------------

47. Give reason(s) for your response …………………………………………………………………………………………

48. Do you intend using family planning service in future? a.Yes [ ] b. No [ ]

49. Any other comment about utilization of family planning among students? ……………………………………………

## **KEYS FOR THE CONTRACEPTIVE ATTITUDE SCALE**

21. Negative

22. Positive

23. Negative

24. Positive

25. Negative

26. Negative

27. Negative

28. Negative

29. Positive

30. Positive

31. Negative

32. Positive

33. Negative

Positive statements:

Strongly disagree=1, strongly agree=5

Negative statements

Strongly disagree=5, strongly agree=1
